# Association study for the role of *MMP8* gene polymorphisms in Colorectal cancer susceptibility

**DOI:** 10.1186/s12885-023-11662-z

**Published:** 2023-11-29

**Authors:** Shuyong Yu, Jiajia Cheng, Ping Li, Le Tian, Zhuang Chen, Zhaowei Chen, Yongyu Li, Jian Song

**Affiliations:** 1 Department of Gastrointestinal Surgery IV, Hainan Cancer Hospital, 570100 Haikou, Hainan China; 2 Department of Digestive Endoscopy, Hainan Cancer Hospital, 570100 Haikou, Hainan China; 3 Department of Gastroenterology, Hainan Cancer Hospital, 570100 Haikou, Hainan China; 4 Department of Gastroenterology, Southern University of Science and Technology Hospital, No. 6019 Liuxian Avenue, Nanshan District, 518000 Shenzhen, Guangdong China

**Keywords:** Colorectal cancer, *MMP8* polymorphisms, Susceptibility, Stratification analysis

## Abstract

**Background:**

Colorectal cancer (CRC) is one of the most common malignant tumors, influenced by several genetic loci in its clinical phenotypes. The aim of this study was to determine the relationship between the *MMP8* gene polymorphism and CRC risk in the Chinese Han population.

**Method:**

This study recruited 688 CRC patients and 690 healthy controls. The relationship between *MMP8* polymorphism and CRC susceptibility was assessed by calculating the odds ratio (OR) and 95% confidence interval (CI) after stratifying by age, gender, body mass index (BMI), smoking, and alcohol consumption under a multi-genetic model.

**Results:**

*MMP8* rs3740938 was associated with increased CRC predisposition (*p* = *0.016*, OR = 1.24, 95% CI: 1.04–1.48), and this association was detected particularly in subjects aged > 60 years, females, people with BMI > 24 kg/m^2^, smokers, and drinkers. Moreover, rs3740938 was found to be associated with the pathological type of rectal cancer.

**Conclusions:**

Our results first displayed that rs3740938 in *MMP8* was a risk factor for CRC predisposition. This finding may provide a new biological perspective for understanding the role of the *MMP8* gene in CRC pathogenesis.

**Supplementary Information:**

The online version contains supplementary material available at 10.1186/s12885-023-11662-z.

## Introduction

Colorectal cancer (CRC) is the third most diagnosed cancer in the world and the second leading cause of cancer death, with a 10.0% incidence and 9.4% mortality [1]. According to Global Cancer Statistics 2020, more than 1.9 million new CRC (including anus) cases and 935,000 deaths were estimated to occur in 2020, representing about one in 10 cancer cases and deaths [1]. According to the National Cancer Center (NCC) of China, CRC is one of the most prevalent cancers, affecting around 408,000 individuals, making up 10% of all cancer cases in the country [2]. In recent decades, the incidence and mortality rates of CRC have been on the rise in recent decades in China [3], and its underlying pathogenesis of CRC remains unclear. While genetic and environmental factors are believed to play a vital role in CRC development [4], several demographic and lifestyle factors such as age, gender, alcohol use, smoking, high body mass index (BMI), and low physical activity have also been reported to be associated with an increased risk of developing CRC and higher mortality rates among patients [5]. At present, many common single-nucleotide polymorphisms (SNPs) associated with CRC risk have been successfully known through genome-wide association studies (GWAS), however, there are still many SNPs susceptible to CRC that have not been identified [6, 7]. Therefore, further investigation into oncogenic SNPs associated with CRC risk is imperative.

*MMP8* (matrix metallopeptidase 8), a member of the MMP family, is an endopeptidase primarily produced by neutrophils. It plays a crucial role in degrading extracellular matrix proteins, growth factors, and cytokines [8]. According to reports, MMP8 is involved in the progression, metastasis, and invasion of cancer through its pro-cancer and anti-tumor functions [9]. For instance, MMP8 has been shown to increase cell-cell adhesion and reduce migration of tongue carcinoma cells by cleaving the anti-adhesive protein FXYD5 [10]. High serum MMP8 levels are associated with reduced survival and systemic inflammation of CRC patients [11]. The enhanced-serum MMP8 level in CRC patients was significantly related to advanced-stage CRC, distant metastasis, lack of MMR, and poor survival [12]. Relevant studies have concluded that the polymorphisms of *MMP8* are associated with the risk of a variety of cancers, including breast cancer [13], thyroid cancer [14], and laryngeal squamous cell carcinoma [15]. Previously, the association between *MMP8* rs11225395 and CRC susceptibility has been reported [16]. The impact of MMP8 genotypes on CRC risk in Taiwan has been explored [17]. The relationship between other loci in *MMP8* and the risk of CRC has not been reported yet.

In previous studies, the association of *MMP8* rs3740938 with the risk of breast cancer has been explored [18]. *MMP8* rs1940475 is associated with the risk of breast cancer [18] and gastric ulcer [19]. *MMP8* rs3765620 is related to ischemic stroke susceptibility [20]. However, the role of these polymorphisms in CRC susceptibility has not been reported. In this case-control study, we selected three variants (rs3740938, rs1940475, and rs3765620) in the exon region of the *MMP8* gene to explore their role in CRC occurrence in the Chinese Han population.

## Methods

### Subjects

To ensure the accuracy and credibility of the research results, before we plan to conduct this study, we used G*power 3.1.9.7 software (https://stats.idre.ucla.edu/other/gpower/) to estimate the sample size. The specific parameters we set are as follows: effect size d = 0.2; α error probability = 0.05; power (1-β err prob) = 95%. This calculation yielded a sample consisting of at least 651 cases and 651 controls. Here, we enrolled a total of 1378 subjects (688 CRC cases and 690 healthy controls) from Hainan Cancer Hospital from 2020 to 2023. The inclusion criteria for CRC cases are: newly diagnosed and histologically confirmed by rectoscopy, endorectal ultrasonography, magnetic resonance imaging (MRI), computed tomography, and histopathological results based on the American Joint Committee on Cancer (AJCC) classification. Patients with a history of cancer or severe chronic diseases were excluded. Prior to any treatment, blood samples were collected from the patient. For the control group, we selected healthy individuals without malignant tumors or digestive diseases from the same hospital as cases, ensuring they were genetically unrelated to CRC patients. All subjects belonged to the Chinese Han ethnicity, and no minors or illiterates were involved in our study. Questionnaires surveys and medical records were used to obtain epidemiological characteristics and pathological data. This research plan was implemented in accordance with the Helsinki Declaration and approved by the Ethics Committee of Hainan Cancer Hospital (No. ZDKJ202008). All subjects provided written informed consent before registration in this study.

### Genotyping

Three SNPs (rs3740938, rs1940475, and rs3765620) in *MMP8* were chosen for analysis. The selection criteria for these SNPs included (1) the dbSNP database with minor allele frequency (MAFs) ≥ 5%; (2) with MassARRAY primer design, a call rate > 99%; (3) and previous association studies [18–20]. Bioinformatics tools such as dbSNP (https://www.ncbi.nlm.nih.gov/snp/), HaploReg v4.1 (https://pubs.broadinstitute.org/mammals/haploreg/haploreg.php), RegulomeDB (https://regulome.stanford.edu/regulome-search/), and QTLbase (http://www.mulinlab.org/qtlbase/index.html) were employed to identify the potentially functional SNPs.

Peripheral whole blood samples (5 mL) were obtained from each participant and stored in tubes containing Ethylene Diamine Tetraacetic Acid (EDTA) anticoagulant. Within 24 h, genomic DNA was isolated using the GoldMag DNA Purification Kit (GoldMag Co. Ltd., Xi’an, China). The DNA samples were quantified using NanoDrop 2000 (Thermo Scientific, Waltham, MA, USA) and stored at − 20 °C. DNA samples with low concentrations or poor quality were excluded from subsequent studies. The MassARRAY platform is based on MALDI-TOF (matrix-assisted laser desorption/ionization—time of flight) mass spectrometry [21, 22]. The analytical accuracy of MALDI-TOF MS is quite high, 0.1–0.01% of the determined mass. Genotyping was performed using the Agena MassARRAY system (Agena, San Diego, CA, USA) with incorporated software (https://www.agenabio.com/). In addition, this study also set up double wells for each sample to ensure the accuracy of the results. For quality control, about 10% of the total samples were chosen randomly and re-genotyped, and the concordance rate reached 100%.

### Statistical data

The demographic data between two groups were tested by student t-test or χ^2^ test for continuous or categorical variables, respectively. The Hardy Weinberg balance (HWE) of the control group was assessed by a goodness-of-fit χ^2^ test. The relationship of *MMP8* polymorphisms with CRC risk was determined by calculating odd ratios (ORs) and 95% confidence intervals (CIs) using a multi-genetic model adjusted for age, sex, BMI, smoking, and alcohol consumption. SNPstats (https://www.snpstats.net/start.htm) was utilized for this analysis. The subgroup analyses were completed within specific subpopulations stratified by age, sex, BMI, smoking, and drinking status. In addition, the impact of genotypes on different pathological types was also evaluated. The false positive reporting probability (FPRP) threshold was set at 0.2 with a prior probability of 0.1, which is used to evaluate the significant association of significant findings [23]. The optimal SNP-SNP interaction model was determined through multifactor dimensionality reduction (MDR) analysis. The data analysis was conducted using SPSS version 18.0 (SPSS Inc., Chicago, Illinois, USA) and MDR version 3.0.2 software. A statistical significance was defined as *p* value < 0.05, and a Bonferroni-corrected *p* < 0.05/3 was considered significance.

## Results

### Subjects characteristics

The case group (59.78 ± 11.29 years) included 402 males and 286 females, and the control group (59.62 ± 9.55 years) consisted of 404 males and 286 females (Table 1). No significant differences between the two groups were found in terms of age (*p* = *0.774*), gender (*p* = *0.964*), smoking (*p* = *0.624*), and drinking (*p* = *0.828*). There was a significant difference in BMI between the two groups (*p* < *0.001*). Among the patient cohort, there were 320 (46.5%) individuals diagnosed with colon cancer and 368 (53.5%) with rectal cancer. Within this group, 183 (26.6%) patients experienced lymph node metastasis, while 263 (38.2%) patients were classified as stage III-IV based on their cancer staging.

**Table 1:** Characteristics of patients with CRC and health controls

Variable		Cases (688)	Control (690)	*p*
Age	Mean ± SD, years	59.78 ± 11.29	59.62 ± 9.55	0.774
	> 60years	353 (51.3%)	380 (55.1%)	
	≤ 60 years	335 (48.7%)	310 (44.9%)	
Gender				0.964
	Male	402 (58.4%)	404 (58.6%)	
	Female	286 (41.6%)	286 (41.4%)	
Smoking				0.624
	Yes	312 (45.3%)	322 (46.7%)	
	No	376 (54.7%)	368 (53.3%)	
Drinking				0.828
	Yes	330 (48.0%)	335 (48.6%)	
	No	358 (52.0%)	355 (51.4%)	
BMI				*< 0.001*
	> 24 kg/m^2^	150 (33.0%)	216 (51.2%)	
	≤ 24 kg/m^2^	304 (67.0%)	206 (48.8%)	
Lymph nodes metastasis				
	Yes	183 (26.6%)		
	No	53 (7.7%)		
	Unavailable	452 (65.7%)		
Pathological type				
	Colon cancer	320 (46.5%)		
	Rectal cancer	368 (53.5%)		
Stage				
	I-II	109 (15.8%)		
	III-IV	263 (38.2%)		
	Unavailable	316 (45.9%)		

CRC, colorectal cancer; BMI, body mass index

*p* values were calculated by χ^2^ test or the Student’s t test

*p* < 0.05 indicates statistical significance

### Relationship of selected variants with CRC risk

Three SNPs (rs3740938, rs1940475, and rs3765620) in *MMP8* were genotyped, and the MAFs of these three SNPs in the two groups were all > 0.05 (Table 2). All HWE *p*–-values for these variants were > 0.05. The results of genotyping displayed that the genotyping success rate of each SNP was > 99.5%. RegulomeDB analysis displayed that rs1940475 was associated with eQTL/caQTL, transcription factor (TF) binding/chromatin accessibility peak. HaploReg v4.1 database displayed that these SNPs might be associated with the regulation of promoter/ enhancer histone marks, DNAse, and /or motif changes. Moreover, the genotypes of rs3740938 (*p* = *0.027*), rs1940475 (*p* = *2.720e-13*), and rs3765620 (*p* = *1.620e-12*) were associated with the expression of *MMP8* in blood. In the allele model, rs3740938 was associated with the higher CRC risk (*p* = *0.016*, OR = 1.24, 95% CI: 1.04–1.48).

**Table 2:** Details of *MMP8* SNPs and the allele model for the association with CRC risk

SNP ID	Chr: Position	Alleles (Ref/Alt)	MAF		HWE			Call rate	OR (95% CI)	*p*	Exon	dbSNP func annot	Haploreg4.1	RegulomeDB	*P*-Value for eQTL in Blood
			Cases	Controls	O(HET)	E(HET)	*p*								
rs3740938	11:102716331	A/G	0.257	0.218	0.326	0.341	0.264	99.8%	1.24 (1.04–1.48)	0.016*	6	Synonymous L (Leu) > L (Leu)	Promoter histone marks; Enhancer histone marks; Motifs changed	other	*0.027*
rs1940475	11:102722517	T/C	0.393	0.367	0.454	0.465	0.514	100.0%	1.12 (0.96–1.30)	0.163	2	Missense K (Lys) > E (Glu)	Enhancer histone marks; DNAse; Motifs changed	eQTL/caQTL + TF binding / chromatin accessibility peak	*2.720e-13*
rs3765620	11:102724761	G/A	0.386	0.359	0.448	0.460	0.508	99.8%	1.13 (0.96–1.31)	0.134	1	Missense T (Thr) > I (Ile)	Enhancer histone marks; DNAse; Motifs changed	other	*1.620e-12*

SNP, Single nucleotide polymorphism; CRC, colorectal cancer; MAF, Minor allele frequency; HWE, Hardy-Weinberg equilibrium; O(HET), Observed heterozygosity frequency; E(HET), Expected heterozygosity frequency

*p* values were calculated from Person’s chi-square test (two-sided)

*p < 0.05 indicates statistical significance. Bold *p* means that the data is statistically significant after Bonferroni correction (*p* < 0.05/3)

dbSNP (https://www.ncbi.nlm.nih.gov/snp/), HaploReg v4.1 (https://pubs.broadinstitute.org/mammals/haploreg/haploreg.php), RegulomeDB (https://regulome.stanford.edu/regulome-search/), QTLbase (http://www.mulinlab.org/qtlbase/index.html)

Genetic model analysis between selected variants and CRC risk was shown in Table 3. *MMP8* rs3740938 contributed to an increased predisposition to CRC under the codominant (*p* = *0.044*, OR = 1.31, 95% CI: 1.04–1.64), dominant (*p* = *0.013*, OR = 1.31, 95% CI: 1.06–1.63) and log-additive (*p* = *0.017*, OR = 1.24, 95% CI: 1.04–1.47) models. The risk-increasing significance of rs3740938 for CRC occurrence still existed after Bonferroni multiple correction (*p* < 0.05/3).

**Table 3:** Effect of *MMP8* variants on the susceptibility to CRC

SNP ID	Model	Genotype	Control	Case	OR (95% CI)	*p*
rs3740938	Codominant	GG	427 (61.9%)	379 (55.2%)	1	0.044*
		GA	225 (32.6%)	261 (38%)	1.31 (1.04–1.64)	
		AA	38 (5.5%)	46 (6.7%)	1.37 (0.87–2.15)	
	Dominant	GG	427 (61.9%)	379 (55.2%)	1	0.013*
		GA-AA	263 (38.1%)	307 (44.8%)	1.31 (1.06–1.63)	
	Recessive	GG-GA	652 (94.5%)	640 (93.3%)	1	0.350
		AA	38 (5.5%)	46 (6.7%)	1.24 (0.79–1.93)	
	Log-additive	---	---	---	1.24 (1.04–1.47)	0.017*
rs1940475	Codominant	CC	280 (40.6%)	261 (37.9%)	1	0.360
		CT	313 (45.4%)	313 (45.5%)	1.08 (0.85–1.35)	
		TT	97 (14.1%)	114 (16.6%)	1.26 (0.92–1.74)	
	Dominant	CC	280 (40.6%)	261 (37.9%)	1	0.310
		CT-TT	410 (59.4%)	427 (62.1%)	1.12 (0.90–1.39)	
	Recessive	CC-CT	593 (85.9%)	574 (83.4%)	1	0.200
		TT	97 (14.1%)	114 (16.6%)	1.21 (0.90–1.63)	
	Log-additive	---	---	---	1.11 (0.96–1.30)	0.170
rs3765620	Codominant	A/A	288 (41.7%)	263 (38.3%)	1	0.330
		G/A	309 (44.8%)	316 (46.1%)	1.12 (0.89–1.41)	
		G/G	93 (13.5%)	107 (15.6%)	1.26 (0.91–1.75)	
	Dominant	A/A	288 (41.7%)	263 (38.3%)	1	0.190
		G/A-G/G	402 (58.3%)	423 (61.7%)	1.15 (0.93–1.43)	
	Recessive	A/A-G/A	597 (86.5%)	579 (84.4%)	1	0.270
		G/G	93 (13.5%)	107 (15.6%)	1.19 (0.88–1.60)	
	Log-additive	---	---	---	1.12 (0.96–1.31)	0.140

CRC, colorectal cancer; SNP, single nucleotide polymorphism; OR, odds ratio; 95% CI, 95% confidence interval; BMI, body mass index

*p* values were calculated by logistic regression analysis with adjustments for age, gender, BMI, smoking and drinking

*p < 0.05 respects the data is statistically significant. Bold *p* means that the data is statistically significant after Bonferroni correction (*p* < 0.05/3)

### Stratification analysis

**Fig. 1:**
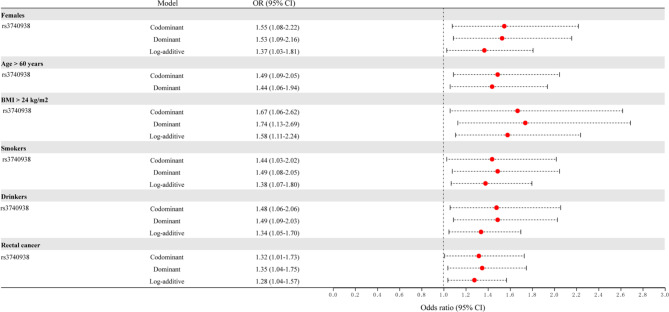
Forest map for the stratification analysis of various confounding factors such as gender, age BMI, smoking, and alcohol consumption and pathological type (rectal cancer)

Stratification analyses by age, gender, BMI, tobacco use, and alcohol were displayed in Fig. 1; Table 4 and Suppl_Table 1. In subjects aged > 60 years, rs3740938 (codominant: *p* = 0.045, OR = 1.49; and dominant: *p* = 0.018, OR = 1.44) might contribute to the increasing CRC risk. After stratification by gender, an association between rs3740938 and an increased CRC susceptibility was observed among females under the dominant (*p* = *0.014*, OR = 1.53) and log-additive (*p* = *0.027*, OR = 1.37) models, and this SNP could be identified as a potential risk marker with a marginal *p* value in codominant model (*p* = 0.048, OR = 1.55). In subjects with BMI > 24 kg/m^2^, rs3740938 conferred to the higher susceptibility to CRC (codominant: *p* = *0.033*, OR = 1.67; dominant: *p* = *0.011*, OR = 1.74; and log-additive: *p* = *0.010*, OR = 1.58). In smokers, rs3740938 might be a -risk-increasing factor for CRC under the codominant (*p* = *0.044*, OR = 1.44), dominant (*p* = *0.015*, OR = 1.49) and log-additive (*p* = *0.014*, OR = 1.38) models. Among drinkers, rs3740938 was associated with the increased CRC susceptibility (codominant: *p* = *0.042*, OR = 1.48; dominant: *p* = *0.012*, OR = 1.49; and log-additive: *p* = *0.018*, OR = 1.34). After Bonferroni multiple correction, the relationships of rs3740938 with CRC susceptibility in females, subjects with BMI > 24 kg/m^2^, smokers and drinkers were also remarkable.

**Table 4:** Stratification for the effect of *MMP8* rs3740938 variant on CRC susceptibility

Model	Genotype	Control	Case	OR (95% CI)	*p* -value	Control	Case	OR (95% CI)	*p* -value
Age stratification		Age > 60 years				Age ≤ 60 years			
Codominant	G/G	239 (62.9%)	191 (54.1%)	1	0.045*	188 (60.6%)	188 (56.5%)	1	0.210
	G/A	117 (30.8%)	140 (39.7%)	1.49 (1.09–2.05)		108 (34.8%)	121 (36.3%)	1.14 (0.82–1.59)	
	A/A	24 (6.3%)	22 (6.2%)	1.17 (0.63–2.16)		14 (4.5%)	24 (7.2%)	1.83 (0.91–3.67)	
Dominant	G/G	239 (62.9%)	191 (54.1%)	1	0.018*	188 (60.6%)	188 (56.5%)	1	0.230
	G/A-A/A	141 (37.1%)	162 (45.9%)	1.44 (1.06–1.94)		122 (39.4%)	145 (43.5%)	1.22 (0.88–1.67)	
Recessive	G/G-G/A	356 (93.7%)	331 (93.8%)	1	0.980	296 (95.5%)	309 (92.8%)	1	0.110
	A/A	24 (6.3%)	22 (6.2%)	1.01 (0.55–1.85)		14 (4.5%)	24 (7.2%)	1.73 (0.87–3.44)	
Log-additive	---	---	---	1.26 (0.99–1.61)	0.056	---	---	1.24 (0.95–1.61)	0.110
**Gender stratification**		**Males**				**Females**			
Codominant	G/G	235 (58.2%)	217 (54.0%)	1	0.450	192 (67.1%)	162 (57.0%)	1	0.048*
	G/A	145 (35.9%)	156 (38.8%)	1.16 (0.86–1.55)		80 (28.0%)	105 (37.0%)	1.55 (1.08–2.22)	
	A/A	24 (5.9%)	29 (7.2%)	1.33 (0.75–2.36)		14 (4.9%)	17 (6.0%)	1.42 (0.68–2.99)	
Dominant	G/G	235 (58.2%)	217 (54.0%)	1	0.240	192 (67.1%)	162 (57.0%)	1	0.014*
	G/A-A/A	169 (41.8%)	185 (46.0%)	1.18 (0.89–1.56)		94 (32.9%)	122 (43.0%)	1.53 (1.09–2.16)	
Recessive	G/G-G/A	380 (94.1%)	373 (92.8%)	1	0.430	272 (95.1%)	267 (94.0%)	1	0.590
	A/A	24 (5.9%)	29 (7.2%)	1.25 (0.71–2.20)		14 (4.9%)	17 (6.0%)	1.22 (0.59–2.54)	
Log-additive	---	---	---	1.16 (0.92–1.45)	0.210	---	---	1.37 (1.03–1.81)	0.027*
**BMI stratification**		**BMI > 24 kg/m** ^**2**^				**BMI ≤ 24 kg/m** ^**2**^			
Codominant	G/G	141 (65.3%)	79 (52.7%)	1	0.033*	131 (63.6%)	179 (59.1%)	1	0.300
	G/A	65 (30.1%)	59 (39.3%)	1.67 (1.06–2.62)		62 (30.1%)	110 (36.3%)	1.29 (0.88–1.90)	
	A/A	10 (4.6%)	12 (8%)	2.25 (0.92–5.48)		13 (6.3%)	14 (4.6%)	0.78 (0.35–1.73)	
Dominant	G/G	141 (65.3%)	79 (52.7%)	1	0.011*	131 (63.6%)	179 (59.1%)	1	0.320
	G/A-A/A	75 (34.7%)	71 (47.3%)	1.74 (1.13–2.69)		75 (36.4%)	124 (40.9%)	1.20 (0.83–1.74)	
Recessive	G/G-G/A	206 (95.4%)	138 (92.0%)	1	0.170	193 (93.7%)	289 (95.4%)	1	0.400
	A/A	10 (4.6%)	12 (80.0%)	1.85 (0.77–4.42)		13 (6.3%)	14 (4.6%)	0.72 (0.33–1.56)	
Log-additive	---	---	---	1.58 (1.11–2.24)	0.010*	---	---	1.08 (0.80–1.46)	0.620
**Smoking stratification**		**Smokers**				**Non-smokers**			
Codominant	G/G	206 (64.0%)	169 (54.3%)	1	0.044*	221 (60.0%)	210 (56.0%)	1	0.470
	G/A	100 (31.1%)	119 (38.3%)	1.44 (1.03–2.02)		125 (34.0%)	142 (37.9%)	1.21 (0.89–1.65)	
	A/A	16 (5.0%)	23 (7.4%)	1.77 (0.90–3.47)		22 (6.0%)	23 (6.1%)	1.10 (0.60–2.04)	
Dominant	G/G	206 (64.0%)	169 (54.3%)	1	0.015*	221 (60.0%)	210 (56.0%)	1	0.240
	G/A-A/A	116 (36.0%)	142 (45.7%)	1.49 (1.08–2.05)		147 (40.0%)	165 (44.0%)	1.19 (0.89–1.60)	
Recessive	G/G-G/A	306 (95.0%)	288 (92.6%)	1	0.200	346 (94.0%)	352 (93.9%)	1	0.930
	A/A	16 (5.0%)	23 (7.4%)	1.54 (0.79–2.99)		22 (6.0%)	23 (6.1%)	1.03 (0.56–1.88)	
Log-additive	---	---	---	1.38 (1.07–1.80)	0.014*	---	---	1.13 (0.89–1.43)	0.320
**Drinking stratification**		**Drinkers**				**Non-drinkers**			
Codominant	G/G	211 (63.0%)	176 (53.5%)	1	0.042*	216 (60.9%)	203 (56.9%)	1	0.600
	G/A	101 (30.1%)	124 (37.7%)	1.48 (1.06–2.06)		124 (34.9%)	137 (38.4%)	1.16 (0.85–1.59)	
	A/A	23 (6.9%)	29 (8.8%)	1.53 (0.85–2.75)		15 (4.2%)	17 (4.8%)	1.20 (0.58–2.47)	
Dominant	G/G	211 (63.0%)	176 (53.5%)	1	0.012*	216 (60.9%)	203 (56.9%)	1	0.310
	G/A-A/A	124 (37.0%)	153 (46.5%)	1.49 (1.09–2.03)		139 (39.1%)	154 (43.1%)	1.17 (0.86–1.58)	
Recessive	G/G-G/A	312 (93.1%)	300 (91.2%)	1	0.330	340 (95.8%)	340 (95.2%)	1	0.740
	A/A	23 (6.9%)	29 (8.8%)	1.32 (0.75–2.35)		15 (4.2%)	17 (4.8%)	1.13 (0.55–2.30)	
Log-additive	---	---	---	1.34 (1.05–1.70)	0.018*	---	---	1.13 (0.88–1.46)	0.330

CRC, colorectal cancer; SNP, single nucleotide polymorphism; OR, odds ratio; 95% CI, 95% confidence interval; BMI, body mass index

*p* values were calculated by logistic regression analysis with adjustments for age, gender, BMI, smoking or drinking

*p < 0.05 respects the data is statistically significant. Bold *p* means that the data is statistically significant after Bonferroni correction (*p* < 0.05/3)

We also explored the correlation of selected SNPs with the pathological types of CRC (Table 5). Stratifying by pathological type, rs3740938 was related to an increased risk of rectal cancer (dominant: *p* = *0.022*, OR = 1.35; and log-additive: *p* = *0.018*, OR = 1.34). Due to lack of information, the correlation of *MMP8* variants with stage and lymph node metastasis in CRC patients has not been explored.

**Table 5:** Association between *MMP8* polymorphisms and the risk of colon cancer and rectal cancer

SNP ID	Model	Genotype	Control	Colon cancer			Rectal cancer		
				N	OR (95% CI)	*p* -value	N	OR (95% CI)	*p* -value
rs3740938	Codominant	G/G	427 (61.9%)	179 (55.9%)	1	0.200	200 (54.6%)	1	0.064
		G/A	225 (32.6%)	122 (38.1%)	1.29 (0.97–1.71)		139 (38.0%)	**1.32 (1.01–1.73)**	
		A/A	38 (5.5%)	19 (5.9%)	1.19 (0.67–2.13)		27 (7.4%)	1.53 (0.90–2.58)	
	Dominant	G/G	427 (61.9%)	179 (55.9%)	1	0.076	200 (54.6%)	1	0.022*
		G/A-A/A	263 (38.1%)	141 (44.1%)	1.28 (0.98–1.67)		166 (45.4%)	**1.35 (1.04–1.75)**	
	Recessive	G/G-G/A	652 (94.5%)	301 (94.1%)	1	0.790	339 (92.6%)	1	0.230
		A/A	38 (5.5%)	19 (5.9%)	1.08 (0.61–1.91)		27 (7.4%)	1.37 (0.82–2.29)	
	Log-additive	---	---	---	1.19 (0.96–1.48)	0.120	---	**1.28 (1.04–1.57)**	0.021*
rs1940475	Codominant	C/C	280 (40.6%)	127 (39.7%)	1	0.790	134 (36.4%)	1	0.240
		C/T	313 (45.4%)	143 (44.7%)	1.01 (0.76–1.35)		170 (46.2%)	1.14 (0.86–1.50)	
		T/T	97 (14.1%)	50 (15.6%)	1.15 (0.77–1.71)		64 (17.4%)	1.38 (0.95–2.01)	
	Dominant	C/C	280 (40.6%)	127 (39.7%)	1	0.750	134 (36.4%)	1	0.180
		C/T-T/T	410 (59.4%)	193 (60.3%)	1.05 (0.80–1.37)		234 (63.6%)	1.19 (0.92–1.55)	
	Recessive	C/C-C/T	593 (85.9%)	270 (84.4%)	1	0.500	304 (82.6%)	1	0.160
		T/T	97 (14.1%)	50 (15.6%)	1.14 (0.78–1.65)		64 (17.4%)	1.29 (0.91–1.82)	
	Log-additive	---	---	---	1.06 (0.87–1.28)	0.570	---	1.17 (0.97–1.40)	0.095
rs3765620	Codominant	A/A	288 (41.7%)	128 (40.0%)	1	0.750	135 (36.9%)	1	0.240
		G/A	309 (44.8%)	144 (45.0%)	1.06 (0.79–1.41)		172 (47.0%)	1.19 (0.90–1.57)	
		G/G	93 (13.5%)	48 (15.0%)	1.17 (0.78–1.76)		59 (16.1%)	1.36 (0.92-2.00)	
	Dominant	A/A	288 (41.7%)	128 (40.0%)	1	0.560	135 (36.9%)	1	0.120
		G/A-G/G	402 (58.3%)	192 (60.0%)	1.08 (0.83–1.42)		231 (63.1%)	1.23 (0.94–1.59)	
	Recessive	A/A-G/A	597 (86.5%)	272 (85.0%)	1	0.510	307 (83.9%)	1	0.240
		G/G	93 (13.5%)	48 (15.0%)	1.14 (0.78–1.66)		59 (16.1%)	1.24 (0.87–1.77)	
	Log-additive	---	---	---	1.08 (0.89–1.30)	0.460	---	1.17 (0.97–1.41)	0.092

SNP, single nucleotide polymorphism; OR, odds ratio; 95% CI, 95% confidence interval

*p* values were calculated by logistic regression analysis with adjustments for age, gender, BMI, smoking or drinking

*p < 0.05 respects the data is statistically significant

### FPRP analysis

Table 6 exhibited the results of the FPRP analysis, with a prior probability level of 0.1 and FPRP of < 0.2, for the positive results. The significant association between rs3740938 and CRC susceptibility remained noteworthy in the overall analysis. And this correlation persisted in females, subjects aged > 60 years, subjects with BMI > 24 kg/m^2^, smokers, and drinkers. Furthermore, the significant association of rs3740938 with susceptibility to rectal cancer remained prominent.

**Table 6:** False-positive report probability for the associations of variants in *MMP* with CRC risk

SNP ID	Model	OR (95% CI)	Prior probability				
			0.25	0.1	0.01	0.001	0.0001
**Overall**							
	Allele	1.24 (1.04–1.48)	**0.049**	**0.134**	0.630	0.945	0.994
	Codominant	1.31 (1.04–1.64)	**0.053**	**0.143**	0.647	0.949	0.995
	Dominant	1.31 (1.06–1.63)	**0.044**	**0.122**	0.605	0.939	0.994
	Log-additive	1.24 (1.04–1.47)	**0.038**	**0.106**	0.567	0.930	0.992
**Females**							
	Codominant	1.55 (1.08–2.22)	**0.052**	**0.141**	0.644	0.948	0.995
	Dominant	1.53 (1.09–2.16)	**0.048**	**0.131**	0.623	0.943	0.994
	Log-additive	1.37 (1.03–1.81)	**0.075**	**0.195**	0.727	0.964	0.996
**Age > 60 years**							
	Codominant	1.49 (1.09–2.05)	**0.043**	**0.118**	0.595	0.937	0.993
	Dominant	1.44 (1.06–1.94)	**0.048**	**0.131**	0.624	0.944	0.994
**BMI > 24 kg/m** ^**2**^							
	Codominant	1.67 (1.06–2.62)	**0.089**	0.227	0.764	0.970	0.997
	Dominant	1.74 (1.13–2.69)	**0.049**	**0.135**	0.631	0.945	0.994
	Log-additive	1.58 (1.11–2.24)	**0.033**	**0.092**	0.527	0.918	0.991
**Smokers**							
	Codominant	1.44 (1.03–2.02)	**0.097**	0.243	0.780	0.973	0.997
	Dominant	1.49 (1.08–2.05)	**0.043**	**0.118**	0.595	0.937	0.993
	Log-additive	1.38 (1.07–1.80)	**0.050**	**0.136**	0.635	0.946	0.994
**Drinkers**							
	Codominant	1.48 (1.06–2.06)	**0.059**	**0.158**	0.674	0.954	0.995
	Dominant	1.49 (1.09–2.03)	**0.034**	**0.096**	0.540	0.922	0.992
	Log-additive	1.34 (1.05–1.70)	**0.046**	**0.125**	0.612	0.941	0.994
**Rectal cancer**							
	Codominant	1.32 (1.01–1.73)	**0.117**	0.285	0.814	0.978	0.998
	Dominant	1.35 (1.04–1.75)	**0.066**	**0.174**	0.699	0.959	0.996
	Log-additive	1.28 (1.04–1.57)	**0.051**	**0.138**	0.638	0.947	0.994

SNP, single nucleotide polymorphism; OR, odds ratio; 95% CI, 95% confidence interval

The level of false-positive report probability threshold was set at 0.2, and Bold represent that noteworthy findings are presented

### MDR analysis

The interaction between these SNPs was evaluated using MDR analysis, and the results were shown in Table 7 and Suppl_Figure 1. Single–locus rs3740938 was the optimal model for evaluating CRC susceptibility (*p* = *0.012*, testing accuracy = 0.5336, cross–validation consistency, 10/10) with the information gain of 0.34%.

**Table 7:** SNP–SNP interaction models of *MMP8* SNPs analyzed by the MDR method

Model	Training Bal. Acc.	Testing Bal. Acc.	CVC	*p*
rs3740938	0.5336	0.5336	10/10	0.012*
rs3740938,rs1940475	0.5340	0.5263	9/10	0.012*
rs3740938,rs1940475,rs3765620	0.5378	0.5270	10/10	0.005*

MDR, multifactor dimensionality reduction; Bal. Acc., balanced accuracy; CVC, cross–validation consistency

*p* values were calculated using χ^2^ tests

*p < 0.05: indicates statistical significance

## Discussion

In this study, we first examined the association of *MMP8* rs3740938 with an increased CRC predisposition in the Chinese Han population. This relationship was particularly significant in subjects aged > 60 years, females, people with BMI > 24 kg/m^2^, smokers and drinkers. Moreover, *MMP8* rs3740938 was related to the pathological type of rectal cancer. These findings contributed valuable data that could potentially be utilized in constructing a genetic panel for predicting CRC risk.

*MMP8* is known to be expressed in various cancer types and may be associated with cancer cell invasion, proliferation, metastasis, and the poor prognosis of cancer patients [9]. MMP8 in serum was identified to be related to CRC patients with bad prognosis [11]. Protein array analysis showed decreased levels of circulating angiogenesis factor MMP8 during treatment with bevacizumab in metastatic CRC [24]. In CRC tissues, YKL-40 is associated with the expression of MMP8 and may be involved in the immunological properties of the tumor microenvironment [25]. MMP8. *MMP8* rs11225395 was connected to the higher CRC risk in a Chinese Han population [16]. However, the contribution of three variants (rs3740938, rs1940475, and rs3765620) in the exon region of *MMP8* gene to CRC occurrence has not been reported. Here, we examined these three SNPs in the *MMP8* gene. Our data displayed a significant association between *MMP8* rs3740938 and an increased CRC predisposition in the Chinese Han population. Bioinformatics analysis using HaploReg v4.1 demonstrated that rs3740938 was related to promoter histone marks, enhancer histone marks and motifs changed. Furthermore, according to the QTLbase database, the genotypes of rs3740938 (*p* = *0.027*) were found to be negatively associated with the expression of *MMP8* in blood. Compared with rs3740938-GG and -GA genotypes, AA genotype may be associated with the lower expression of *MMP8* mRNA. These findings suggested that the role of rs3740938 in CRC may be through affecting gene expression of *MMP8*, thereby affecting CRC occurrence. However, further experimental confirmation is needed to validate this hypothesis.

The incidence and mortality rates related to CRC have shown a steady increase [26]. The incidence and mortality of CRC are often higher in men than in women [27]. Sex hormones are considered to be the factors leading to gender differences in the incidence and mortality of CRC [28]. Here, the relationship of *MMP8* polymorphisms with CRC occurrence under the stratified analysis by age, sex and BMI was explored. *MMP8* rs3740938 might contribute to an increased susceptibility to CRC in participants aged > 60 years, females, and people with BMI > 24 kg/m^2^, indicating that the effects of rs3740938 on CRC occurrence are specific to age, gender, and BMI. As is well known, smoking increases the risk of various cancers, such as lung cancer, head and neck cancer, stomach cancer, etc [29]. It is a significant risk factor for CRC, with a dose-dependent relationship where the risk increases with the intensity and duration of smoking [30]. For a long time, alcohol consumption has been shown to be associated with the development of CRC and is considered as a crucial targeted factor related to the adverse consequences of CRC [31]. We also explored the correlation of selected SNPs with cigarette and alcohol in relation to CRC occurrence, and our results indicated that rs3740938 might act as an increasing-risk factor for CRC in smokers and drinkers. Additionally, rs3740938 was also observed to be related to an increased risk of rectal cancer. Therefore, according to our research results, this locus can be identified as a key research object for further investigation into CRC risk.

There are also potential limitations to our research. First, the sample size of our study was limited, and all participants were Chinese Han people. Future studies will involve a larger and more diverse sample population, along with validation experiments to confirm our results. Second, the lack of comprehensive information on various factors such as environmental exposure, lifestyle choices, and clinicopathological data (including cancer progression, metastasis, and invasion) hinders a thorough understanding of their role in the association between the selected SNPs and CRC risk. Therefore, further studies is required to investigate these aspects and collected complete clinicopathological data to evaluate the relationship accurately. Third, the potential mechanisms and functions of these SNPs in relation to CRC risk, including association of high serum *MMP8* levels with*MMP8* SNPs have not been fully elucidated. In subsequent researches, we will design detailed experiments to explore the expression data of *MMP8* and the potential mechanisms and functions of these SNPs in CRC. Fourth, our study only assesses the correlation between three SNPs in the exon region of the *MMP8* gene and CRC risk, and a large number of exonic or intronic variants remain to be studied. In subsequent studies, we will further explore the association of other loci in *MMP8* with CRC susceptibility. Despite the above limitations, this is the first study that has reported *MMP8* rs3740938 was associated with the increased CRC predisposition in a Chinese Han population, and this variant could serve as potential biomarkers of CRC susceptibility. These findings increased our knowledge regarding the effect of *MMP8* on the process of CRC occurrence, provided some data for future explorations of the relationship between *MMP8* and CRC risk in different populations, and also helped to establish new warning and treatment methods for CRC in futures studies. In the next step, we will further explore the functions of these SNPs based on the results of this study, in order to provide new theoretical basis and targets for the diagnosis and treatment of CRC.

## Conclusion

Our results show that *MMP8* rs3740938 might be a risk-increasing factor for CRC, revealing for the first time the role of rs3740938 in *MMP8* in CRC risk among the Chinese Han population. Our findings might provide new biological insights into the role of *MMP8* gene in the formation and progression of CRC.

### Electronic supplementary material

Below is the link to the electronic supplementary material.

**Supplementary Table 1**. Stratification for the effect of MMP8 rs1940475 and rs3765620 variants on CRC susceptibility.

Supplementary Material 2

## Data Availability

The datasets generated and/or analysed during the current study are available in the Zenodo repository, https://zenodo.org/records/10012137.
