# Supplementary material for: Association study for the role of MMP8 gene polymorphisms in Colorectal cancer susceptibility
Source: BMC Cancer. 2023 Nov 29;23:1169. doi: 10.1186/s12885-023-11662-z (PMC10688471; doi:10.1186/s12885-023-11662-z)
Supplement: Supplementary file 1 — Supplementary Table 1. Stratification for the effect of MMP8 rs1940475 and rs3765620 variants on CRC susceptibility. [file 12885_2023_11662_MOESM1_ESM.docx]

Suppl_Table 1. Stratification for the effect of *MMP8* **rs1940475 and rs3765620** variants on CRC susceptibility

| SNP ID | **Model** | | | **Genotype** | **control** | **case** | **OR (95% CI)** | ***p* -value** | **control** | **case** | **OR (95% CI)** | ***p* -value** |
| --- | --- | --- | --- | --- | --- | --- | --- | --- | --- | --- | --- | --- |
| Age stratification | | |  | | **Age > 60 years** | | | | **Age ≤ 60 years** | | | |
| rs1940475 | Codominant | | | C/C | 156 (41%) | 126 (35.7%) | 1 | 0.280 | 124 (40%) | 135 (40.3%) | 1 | 0.510 |
|  |  |  |  | C/T | 165 (43.4%) | 163 (46.2%) | 1.24 (0.89-1.71) |  | 148 (47.7%) | 150 (44.8%) | 0.93 (0.67-1.30) |  |
|  |  |  |  | T/T | 59 (15.5%) | 64 (18.1%) | 1.36 (0.88-2.09) |  | 38 (12.3%) | 50 (14.9%) | 1.24 (0.76-2.02) |  |
|  | Dominant | | | C/C | 156 (41.0%) | 126 (35.7%) | 1 | 0.120 | 124 (40.0%) | 135 (40.3%) | 1 | 0.970 |
|  |  |  |  | C/T-T/T | 224 (59.0%) | 227 (64.3%) | 1.27 (0.94-1.72) |  | 186 (60.0%) | 200 (59.7%) | 0.99 (0.72-1.36) |  |
|  | Recessive | | | C/C-C/T | 321 (84.5%) | 289 (81.9%) | 1 | 0.340 | 272 (87.7%) | 285 (85.1%) | 1 | 0.280 |
|  |  |  |  | T/T | 59 (15.5%) | 64 (18.1%) | 1.21 (0.82-1.79) |  | 38 (12.3%) | 50 (14.9%) | 1.29 (0.81-2.03) |  |
|  | Log-additive | | | --- | --- | --- | 1.18 (0.96-1.45) | 0.120 | --- | --- | 1.06 (0.85-1.33) | 0.610 |
| rs3765620 | Codominant | | | A/A | 161 (42.4%) | 126 (35.7%) | 1 | 0.160 | 127 (41.0%) | 137 (41.1%) | 1 | 0.640 |
|  |  |  |  | G/A | 165 (43.4%) | 169 (47.9%) | 1.33 (0.96-1.83) |  | 144 (46.5%) | 147 (44.1%) | 0.95 (0.68-1.33) |  |
|  |  |  |  | G/G | 54 (14.2%) | 58 (16.4%) | 1.38 (0.89-2.15) |  | 39 (12.6%) | 49 (14.7%) | 1.19 (0.73-1.95) |  |
|  | Dominant | | | A/A | 161 (42.4%) | 126 (35.7%) | 1 | 0.058 | 127 (41%) | 137 (41.1%) | 1 | 0.990 |
|  |  |  |  | G/A-G/G | 219 (57.6%) | 227 (64.3%) | 1.34 (0.99-1.81) |  | 183 (59.0%) | 196 (58.9%) | 1.00 (0.73-1.37) |  |
|  | Recessive | | | A/A-G/A | 326 (85.8%) | 295 (83.6%) | 1 | 0.410 | 271 (87.4%) | 284 (85.3%) | 1 | 0.380 |
|  |  |  |  | G/G | 54 (14.2%) | 58 (16.4%) | 1.19 (0.79-1.79) |  | 39 (12.6%) | 49 (14.7%) | 1.23 (0.78-1.94) |  |
|  | Log-additive | | | --- | --- | --- | 1.21 (0.98-1.49) | 0.081 | --- | --- | 1.05 (0.84-1.32) | 0.660 |
| **Gender** stratification | |  | | | **Males** | | | | **Females** | | | |
| rs1940475 | Codominant | | | C/C | 157 (38.9%) | 151 (37.6%) | 1 | 0.690 | 123 (43%) | 110 (38.5%) | 1 | 0.420 |
|  |  |  |  | C/T | 185 (45.8%) | 180 (44.8%) | 1.00 (0.74-1.36) |  | 128 (44.8%) | 133 (46.5%) | 1.17 (0.82-1.67) |  |
|  |  |  |  | T/T | 62 (15.3%) | 71 (17.7%) | 1.18 (0.78-1.77) |  | 35 (12.2%) | 43 (15.0%) | 1.39 (0.83-2.34) |  |
|  | Dominant | | | C/C | 157 (38.9%) | 151 (37.6%) | 1 | 0.760 | 123 (43%) | 110 (38.5%) | 1 | 0.260 |
|  |  |  |  | C/T-T/T | 247 (61.1%) | 251 (62.4%) | 1.05 (0.79-1.39) |  | 163 (57%) | 176 (61.5%) | 1.22 (0.87-1.70) |  |
|  | Recessive | | | C/C-C/T | 342 (84.7%) | 331 (82.3%) | 1 | 0.390 | 251 (87.8%) | 243 (85.0%) | 1 | 0.320 |
|  |  |  |  | T/T | 62 (15.3%) | 71 (17.7%) | 1.18 (0.81-1.71) |  | 35 (12.2%) | 43 (15.0%) | 1.28 (0.79-2.07) |  |
|  | Log-additive | | | --- | --- | --- | 1.07 (0.88-1.30) | 0.500 | --- | --- | 1.18 (0.92-1.50) | 0.190 |
| rs3765620 | Codominant | | | A/A | 163 (40.4%) | 152 (37.9%) | 1 | 0.560 | 125 (43.7%) | 111 (39.0%) | 1 | 0.490 |
|  |  |  |  | G/A | 184 (45.5%) | 182 (45.4%) | 1.05 (0.77-1.42) |  | 125 (43.7%) | 134 (47.0%) | 1.22 (0.85-1.74) |  |
|  |  |  |  | G/G | 57 (14.1%) | 67 (16.7%) | 1.25 (0.83-1.90) |  | 36 (12.6%) | 40 (14.0%) | 1.25 (0.74-2.11) |  |
|  | Dominant | | | A/A | 163 (40.4%) | 152 (37.9%) | 1 | 0.530 | 125 (43.7%) | 111 (39.0%) | 1 | 0.230 |
|  |  |  |  | G/A-G/G | 241 (59.6%) | 249 (62.1%) | 1.10 (0.83-1.46) |  | 161 (56.3%) | 174 (61%) | 1.23 (0.88-1.71) |  |
|  | Recessive | | | A/A-G/A | 347 (85.9%) | 334 (83.3%) | 1 | 0.300 | 250 (87.4%) | 245 (86.0%) | 1 | 0.630 |
|  |  |  |  | G/G | 57 (14.1%) | 67 (16.7%) | 1.22 (0.83-1.80) |  | 36 (12.6%) | 40 (14.0%) | 1.13 (0.69-1.84) |  |
|  | Log-additive | | | --- | --- | --- | 1.10 (0.91-1.35) | 0.330 | --- | --- | 1.15 (0.90-1.46) | 0.270 |
| BMI stratification | | |  | | **BMI > 24 kg/m^2^** | | | | **BMI ≤ 24 kg/m^2^** | | | |
| rs1940475 | Codominant | | | C/C | 92 (42.6%) | 55 (36.7%) | 1 | 0.470 | 83 (40.3%) | 122 (40.1%) | 1 | 0.990 |
|  |  |  |  | C/T | 92 (42.6%) | 71 (47.3%) | 1.31 (0.83-2.08) |  | 95 (46.1%) | 142 (46.7%) | 1.02 (0.70-1.50) |  |
|  |  |  |  | T/T | 32 (14.8%) | 24 (16.0%) | 1.30 (0.69-2.45) |  | 28 (13.6%) | 40 (13.2%) | 0.99 (0.57-1.75) |  |
|  | Dominant | | | C/C | 92 (42.6%) | 55 (36.7%) | 1 | 0.220 | 83 (40.3%) | 122 (40.1%) | 1 | 0.920 |
|  |  |  |  | C/T-T/T | 124 (57.4%) | 95 (63.3%) | 1.31 (0.85-2.02) |  | 123 (59.7%) | 182 (59.9%) | 1.02 (0.71-1.46) |  |
|  | Recessive | | | C/C-C/T | 184 (85.2%) | 126 (84.0%) | 1 | 0.700 | 178 (86.4%) | 264 (86.8%) | 1 | 0.940 |
|  |  |  |  | T/T | 32 (14.8%) | 24 (16.0%) | 1.12 (0.63-2.00) |  | 28 (13.6%) | 40 (13.2%) | 0.98 (0.58-1.66) |  |
|  | Log-additive | | | --- | --- | --- | 1.18 (0.87-1.59) | 0.290 | --- | --- | 1.00 (0.77-1.31) | 0.970 |
| rs3765620 | Codominant | | | A/A | 94 (43.5%) | 55 (36.9%) | 1 | 0.390 | 84 (40.8%) | 124 (40.8%) | 1 | 0.960 |
|  |  |  |  | G/A | 91 (42.1%) | 72 (48.3%) | 1.37 (0.87-2.17) |  | 95 (46.1%) | 143 (47%) | 1.03 (0.70-1.50) |  |
|  |  |  |  | G/G | 31 (14.3%) | 22 (14.8%) | 1.25 (0.66-2.39) |  | 27 (13.1%) | 37 (12.2%) | 0.95 (0.54-1.68) |  |
|  | Dominant | | | A/A | 94 (43.5%) | 55 (36.9%) | 1 | 0.180 | 84 (40.8%) | 124 (40.8%) | 1 | 0.960 |
|  |  |  |  | G/A-G/G | 122 (56.5%) | 94 (63.1%) | 1.34 (0.87-2.07) |  | 122 (59.2%) | 180 (59.2%) | 1.01 (0.70-1.45) |  |
|  | Recessive | | | A/A-G/A | 185 (85.7%) | 127 (85.2%) | 1 | 0.860 | 179 (86.9%) | 267 (87.8%) | 1 | 0.810 |
|  |  |  |  | G/G | 31 (14.3%) | 22 (14.8%) | 1.06 (0.58-1.92) |  | 27 (13.1%) | 37 (12.2%) | 0.94 (0.55-1.60) |  |
|  | Log-additive | | | --- | --- | --- | 1.18 (0.87-1.59) | 0.300 | --- | --- | 0.99 (0.76-1.29) | 0.940 |
| Smoking stratification | | |  | | **Smokers** | | | | **Non-smokers** | | | |
| rs1940475 | Codominant | | | C/C | 123 (38.2%) | 110 (35.3%) | 1 | 0.170 | 157 (42.7%) | 151 (40.2%) | 1 | 0.760 |
|  |  |  |  | C/T | 157 (48.8%) | 144 (46.1%) | 1.02 (0.72-1.44) |  | 156 (42.4%) | 169 (45.0%) | 1.12 (0.82-1.54) |  |
|  |  |  |  | T/T | 42 (13.0%) | 58 (18.6%) | 1.54 (0.95-2.47) |  | 55 (14.9%) | 56 (14.9%) | 1.06 (0.69-1.64) |  |
|  | Dominant | | | C/C | 123 (38.2%) | 110 (35.3%) | 1 | 0.470 | 157 (42.7%) | 151 (40.2%) | 1 | 0.490 |
|  |  |  |  | C/T-T/T | 199 (61.8%) | 202 (64.7%) | 1.13 (0.82-1.56) |  | 211 (57.3%) | 225 (59.8%) | 1.11 (0.83-1.49) |  |
|  | Recessive | | | C/C-C/T | 280 (87.0%) | 254 (81.4%) | 1 | 0.058 | 313 (85.0%) | 320 (85.1%) | 1 | 0.999 |
|  |  |  |  | T/T | 42 (13.0%) | 58 (18.6%) | 1.52 (0.98-2.34) |  | 55 (14.9%) | 56 (14.9%) | 1.00 (0.67-1.50) |  |
|  | Log-additive | | | --- | --- | --- | 1.19 (0.95-1.49) | 0.130 | --- | --- | 1.05 (0.86-1.29) | 0.630 |
| rs3765620 | Codominant | | | A/A | 126 (39.1%) | 112 (35.9%) | 1 | 0.260 | 162 (44.0%) | 151 (40.4%) | 1 | 0.560 |
|  |  |  |  | G/A | 156 (48.5%) | 147 (47.1%) | 1.06 (0.75-1.49) |  | 153 (41.6%) | 169 (45.2%) | 1.19 (0.87-1.62) |  |
|  |  |  |  | G/G | 40 (12.4%) | 53 (17.0%) | 1.49 (0.91-2.42) |  | 53 (14.4%) | 54 (14.4%) | 1.09 (0.70-1.70) |  |
|  | Dominant | | | A/A | 126 (39.1%) | 112 (35.9%) | 1 | 0.420 | 162 (44.0%) | 151 (40.4%) | 1 | 0.310 |
|  |  |  |  | G/A-G/G | 196 (60.9%) | 200 (64.1%) | 1.14 (0.83-1.58) |  | 206 (56.0%) | 223 (59.6%) | 1.16 (0.87-1.56) |  |
|  | Recessive | | | A/A-G/A | 282 (87.6%) | 259 (83.0%) | 1 | 0.110 | 315 (85.6%) | 320 (85.6%) | 1 | 0.990 |
|  |  |  |  | G/G | 40 (12.4%) | 53 (17.0%) | 1.44 (0.92-2.25) |  | 53 (14.4%) | 54 (14.4%) | 1.00 (0.67-1.51) |  |
|  | Log-additive | | | --- | --- | --- | 1.18 (0.94-1.48) | 0.160 | --- | --- | 1.08 (0.88-1.33) | 0.470 |
| Drinking stratification | | |  | | **Drinkers** | | | | **Non-drinkers** | | | |
| rs1940475 | Codominant | | | C/C | 134 (40.0%) | 125 (37.9%) | 1 | 0.230 | 146 (41.1%) | 136 (38%) | 1 | 0.700 |
|  |  |  |  | C/T | 156 (46.6%) | 145 (43.9%) | 1.01 (0.72-1.41) |  | 157 (44.2%) | 168 (46.9%) | 1.15 (0.83-1.58) |  |
|  |  |  |  | T/T | 45 (13.4%) | 60 (18.2%) | 1.45 (0.92-2.29) |  | 52 (14.7%) | 54 (15.1%) | 1.10 (0.70-1.73) |  |
|  | Dominant | | | C/C | 134 (40.0%) | 125 (37.9%) | 1 | 0.530 | 146 (41.1%) | 136 (38.0%) | 1 | 0.410 |
|  |  |  |  | C/T-T/T | 201 (60.0%) | 205 (62.1%) | 1.10 (0.81-1.51) |  | 209 (58.9%) | 222 (62.0%) | 1.13 (0.84-1.53) |  |
|  | Recessive | | | C/C-C/T | 290 (86.6%) | 270 (81.8%) | 1 | 0.087 | 303 (85.3%) | 304 (84.9%) | 1 | 0.910 |
|  |  |  |  | T/T | 45 (13.4%) | 60 (18.2%) | 1.44 (0.95-2.20) |  | 52 (14.7%) | 54 (15.1%) | 1.02 (0.68-1.55) |  |
|  | Log-additive | | | --- | --- | --- | 1.16 (0.93-1.44) | 0.190 | --- | --- | 1.07 (0.87-1.32) | 0.520 |
| rs3765620 | Codominant | | | A/A | 140 (41.8%) | 124 (37.7%) | 1 | 0.290 | 148 (41.7%) | 139 (38.9%) | 1 | 0.770 |
|  |  |  |  | G/A | 149 (44.5%) | 147 (44.7%) | 1.13 (0.81-1.58) |  | 160 (45.1%) | 169 (47.3%) | 1.12 (0.82-1.54) |  |
|  |  |  |  | G/G | 46 (13.7%) | 58 (17.6%) | 1.44 (0.91-2.27) |  | 47 (13.2%) | 49 (13.7%) | 1.10 (0.69-1.75) |  |
|  | Dominant | | | A/A | 140 (41.8%) | 124 (37.7%) | 1 | 0.250 | 148 (41.7%) | 139 (38.9%) | 1 | 0.470 |
|  |  |  |  | G/A-G/G | 195 (58.2%) | 205 (62.3%) | 1.20 (0.88-1.64) |  | 207 (58.3%) | 218 (61.1%) | 1.12 (0.83-1.51) |  |
|  | Recessive | | | A/A-G/A | 289 (86.3%) | 271 (82.4%) | 1 | 0.160 | 308 (86.8%) | 308 (86.3%) | 1 | 0.880 |
|  |  |  |  | G/G | 46 (13.7%) | 58 (17.6%) | 1.35 (0.88-2.06) |  | 47 (13.2%) | 49 (13.7%) | 1.03 (0.67-1.59) |  |
|  | Log-additive | | | --- | --- | --- | 1.18 (0.95-1.47) | 0.130 | --- | --- | 1.07 (0.86-1.33) | 0.550 |

CRC, colorectal cancer; SNP, single nucleotide polymorphism; OR, odds ratio; 95% CI, 95% confidence interval; BMI, body mass index.

*p* values were calculated by logistic regression analysis with adjustments for age, gender, BMI, smoking or drinking.
