# Supplementary figures and images for: Association study for the role of MMP8 gene polymorphisms in Colorectal cancer susceptibility
Source: BMC Cancer. 2023 Nov 29;23:1169. doi: 10.1186/s12885-023-11662-z (PMC10688471; doi:10.1186/s12885-023-11662-z)

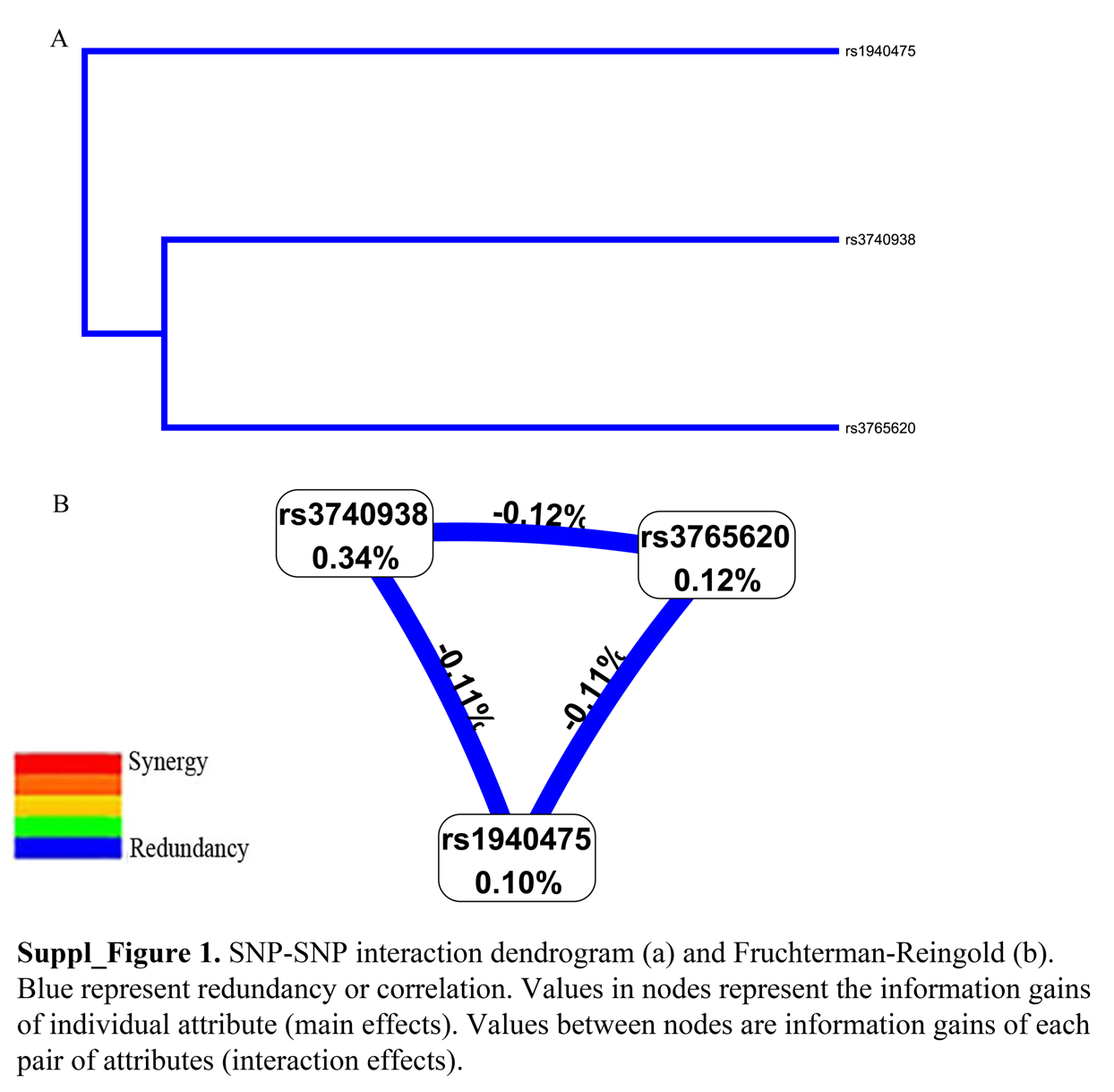

Supplement: Supplementary file 2 — Supplementary Material 2 [file 12885_2023_11662_MOESM2_ESM.png]
